# Human embryos cultured *in vitro* to 14 days

**DOI:** 10.1098/rsob.170003

**Published:** 2017-01-25

**Authors:** Samantha A. Morris

**Affiliations:** 1 Department of Developmental Biology, Washington University School of Medicine in St. Louis, 660 S. Euclid Avenue, Campus Box 8103, St. Louis, MO 63110, USA; 2 Department of Genetics, Center of Regenerative Medicine, Washington University School of Medicine in St. Louis, 660 S. Euclid Avenue, Campus Box 8103, St. Louis, MO 63110, USA

**Keywords:** mammalian embryo, development, cell fate

## Abstract

We know a great deal about the development of the mammalian embryo until the time that the blastocyst implants into the uterus. With model organisms such as the mouse, we have also developed a considerable understanding of development immediately around gastrulation as embryos can be recovered at this stage for short-term *in vitro* culture. However, the intervening period of development remained a ‘black box’ because it takes place as the blastocyst is implanting into the uterus. Over the past 6 years, techniques pioneered and developed in Magdalena Zernicka-Goetz's laboratory for the *in vitro* culture of embryos through these implantation stages have opened up this box, affording the first glimpse of embryonic development through these previously hidden stages. Remarkably, the techniques developed with mouse embryos are equally applicable to human embryos, ushering the very first opportunities for studying our own development throughout this time. Here, I outline how the culture methods were developed, paving the way to culture of the human embryo to the point of gastrulation, an accomplishment recognized as the People's Choice for the *Scientific Breakthrough of 2016* in Science magazine. I also discuss the new ethical challenges raised by the possibility of extending the time limits for human embryo culture.

Our quite detailed understanding of the early stages of mammalian embryonic development reflects the fact that we have for many decades been able to culture embryos to the point of implantation. Such studies have focused on the mouse as an experimental organism where, as in all vertebrates, embryonic development requires repeated rounds of cleavage divisions. These progressively divide the single-cell zygote into the hundred or so cells of the blastocyst over about 4.5 days in the case of the mouse preimplantation embryo. This period represents a critical stage in development when the first three tissue types of the embryo are formed. This happens after the 8-cell stage when cells compact, become polarized, and then undertake asymmetric divisions. These divisions generate polarized cells that remain on the outside and generate trophectoderm (TE), that will later mainly contribute to the placenta, and apolar cells that will become the inner cell mass (ICM). The first ICM cells to be produced in the fourth round of cleavage will contribute mainly to the pluripotent epiblast (EPI), which will generate all of the embryo proper, and those produced in the fifth cleavage round will generate mainly primitive endoderm (PE), a second extra-embryonic tissue [1–3]. The resulting morula then cavitates to form a blastocyst. When the cavity is fully expanded, the blastocyst hatches out of its egg-shell-like zona pellucida and implants through the invasion of TE cells into the endometrium of the uterus. Other TE cells develop adjacent to the EPI as the extra-embryonic ectoderm (ExE) to form a cup-shaped structure, the egg cylinder, enveloped by visceral endoderm (VE) which is descended from the PE (figure 1). It is during the development of this cup-like structure that the foundations of the mouse body plan are laid.

**Figure 1.**
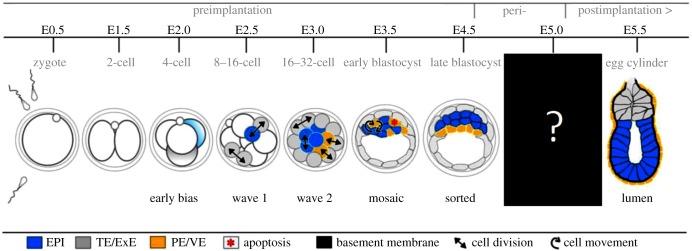
Pre- and postimplantation development of the mouse embryo. The events of implantation were hidden in a black box. Taken from Bedzhov *et al*. [5].

By studying snap-shot images of fixed embryos at successive stages, a picture has emerged of how the body plan becomes established but the picture is incomplete and lacks detail. It has been clear from these studies, however, that the extra-embryonic tissues of the egg cylinder play an important role in signalling the development of the adjacent EPI. A cluster of VE cells at the distal tip of the egg cylinder, the distal VE (DVE), begins to express anterior markers and a subset migrates up one side of the cup of EPI to form an anterior signalling centre, the anterior VE (AVE) [6–8]. Similarly, the ExE abutting the EPI is a source of a BMP4 signal that specifies posterior development on the lip of the EPI cup opposite the AVE [9–12]. This posterior part identifies the site of gastrulation that emerges in response to Wnt signalling, preceding the expression of T/Brachyury and other mesoderm markers [13].

However, precisely how these signalling centres arise has been very difficult to analyse, because the process takes place as the embryo implants into the uterus. In 2012, the goal of finding the origins of the anterior signalling cells motivated the Zernicka-Goetz group, which I was part of, to establish an *in vitro* culture system permitting the step-wise development of the AVE to be followed in real time [14]. *In vitro* culture of embryos through the implantation stages had been attempted in the 1970s with limited success [15–17], but it was crucial to build on this work in a way that would permit state-of-the-art imaging of development. Two factors proved crucial: first, to supplement the media with serum obtained from human umbilical cords; second, to provide the embryos with a polyacrylamide hydrogel substrate of suitable stiffness coated with proteins of which collagen was key. Using these conditions, about 80% of embryos attached, with the TE spreading out onto the substrate. Around half of these embryos developed into structures resembling egg cylinders that recapitulated the same spatial patterns of expression of the respective ExE, VE and EPI marker genes, *Cdx2, Gata4* and *Oct4*, as naturally developing embryos. By following expression of *Cerl-GFP* as a marker for the origins of the AVE, a small cluster of cells was identified as blastocysts flattened onto the matrix that became consolidated as the egg cylinder emerged. The most anteriorly located cell in the cluster showed the strongest *Cerl-GFP* expression and led the anterior migration. Ablation of such leading cells prevented AVE migration, pointing to their importance in the correct establishment of the anterior–posterior axis [14].

These early experiments highlighted a need for a more careful examination of the cellular events as the EPI becomes reorganized during embryo implantation. Doing so led to a completely new understanding of the nature of the morphological changes undertaken by the EPI as the blastocyst implants [18]. These findings were possible through further optimization of the *in vitro* culture system to enable development of zona-free blastocysts seeded directly onto microscopy-grade plastic microplates to facilitate time-lapse microscopy. It was also necessary to modify the media to overcome the batch variations between isolates of human cord serum. Blastocysts were plated in IVC1, the medium originally described by Morris *et al*. [14], but once they had attached, this was switched to IVC2 modified by the substitution of KnockOut Serum Replacement for human cord serum and also supplemented with β-oestradiol, progesterone, *N*-acetyl-l-cysteine, and an Insulin-Transferrin-Selenium-Ethanolamine cocktail [18].

Using this system, it soon became clear that the textbook description of pro-amniotic cavity formation at this stage was incorrect. This textbook model had emerged based on observations that the cavitation of embryoid bodies, formed from aggregates of embryonic stem (ES) cells or embryonal carcinoma cells, occurs through apoptosis. These findings led to the suggestion that the EPI cavity was formed in the same way in the embryo [19,20]. This model proposes that the VE is the source of a signal for programmed cell death in the EPI, and also posits that a second signal for survival is supplied to those EPI cells directly contacting the surrounding basal membrane. In concert, these signals result in apoptosis of only the EPI cell core. Contrary to this long-standing model, there was no evidence of cell death as the cavity formed in embryos developing in this modified system. Moreover, chemically inhibiting apoptosis or preventing it by removing p53 had no effect on cavitation [18]. Instead, the EPI was observed to become reorganized into a rosette-like structure of highly polarized cells, followed by the formation of a central lumen via hollowing of their apical membranes (figure 2). In contrast to the hundreds of cells seen in ES cell aggregates, the EPI cells of the blastocyst became arranged into rosettes of an average of 12–13 cells that underwent two rounds of cell division over the next 12 h. E-cadherin and F-actin were localized on the apical site of the wedge-shaped cells, with this shape change brought about through actinomyosin-mediated constriction close to the adherance junction. Lumenogenesis required separation of the apical membranes through charge repulsion likely to be mediated by anti-adhesive molecules such as podocalyxin, where the signal for this process appeared to be a polarization cue arising from the basal membrane. It emerged that molecules of the extracellular matrix (ECM), supplied via Matrigel, could substitute for the basal membrane and induce isolated EPI cells to polarize and begin to cavitate. This led Bedzhov & Zernicka-Goetz [18] to demonstrate that a small number of ES cells were able to recapitulate rosette formation if cultured within a three-dimensional scaffold of Matrigel. However, β1-integrin −/− ES cells were unable to form rosettes in this manner. Thus, the study uncovered a previously hidden chain of morphogenetic events by which the ECM triggers self-organization of the embryo's stem cells in a β1-integrin-mediated signalling process.

**Figure 2.**
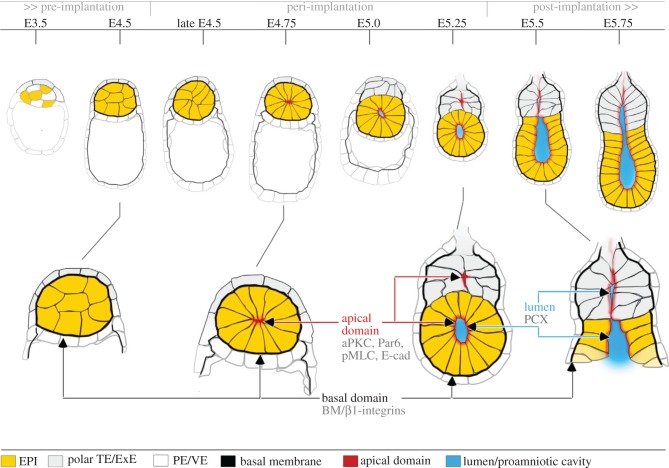
A new model of peri-implantation development in the mouse. *In vitro* culture has revealed the rosette of polarized EPI cells that forms upon implantation and which is required for lumenogenesis. Taken from Bedzhov & Zernicka-Goetz [18].

The protocol used in these studies has proved to be very robust. The Zernicka-Goetz lab over recent years developed several variations on the method showing not only that blastocysts could attach to the substrate after removal of the zona, but that it was also possible to surgically remove part of the TE whereupon egg cylinder structures were generated more efficiently, with very little developmental lag upon implanting [4,5]. Moreover, egg cylinders could also develop as free floating embryos cultured in hanging drops, indicating that physical contact with the substrate, or by inference with the uterus in natural development, is not required for the self-organization of the egg cylinder [21].

Following the establishment of this robust protocol of mouse embryo culture through implantation stages, the Zernicka-Goetz group began work to apply the technique to human embryos, with great success. Considering current efforts in research to increase rigour and reproducibility, Zernicka-Goetz and colleagues should be commended for their efforts to ensure that their high-impact findings were replicated. For example, they shared their initial mouse techniques via provision of transparent protocols [4,5]. Furthermore, in the very early stages of the application of their technique to human embryos, they instructed an independent laboratory, sharing the procedure with Alessia Deglincerti of the Brivanlou lab at Rockefeller University, ensuring that their findings could be more widely reproduced. This enabled both the Cambridge and Rockefeller groups to make the remarkable achievement of culturing human embryos to the point of gastrulation, 14 days from their time of fertilization [22,23].

The success of this technique opens a wealth of opportunity. If our knowledge of mouse embryonic development through implantation stages has been restricted, then what of the human embryo where extremely few studies have been based on fixed specimens? Attempts were previously made to co-culture human blastocysts and endometrial cells (e.g. [24]) but the extent to which these methods could recapitulate human embryo development was an open question. The two new studies showed that just as with the mouse, the human embryo possesses an intrinsic capacity for self-organization, without the participation of maternal tissues [22,23]. Despite the similar appearance of their blastocysts, mouse and human embryos differ substantially in their morphology and instead of the cup-like egg cylinder, the EPI of the human embryo is flattened into a bilaminar disc. The studies of the two groups showed that human embryos developing *in vitro* possess all the key landmarks of normal development. These include segregation of the pluripotent embryonic and extra-embryonic lineages; the morphogenetic rearrangements leading to generation of the bilaminar disc; the formation of a pro-amniotic cavity within the embryonic lineage that in human embryos splits the pluripotent cells into two populations corresponding to the founders of the embryo proper and the amniotic epithelium; the formation of the prospective yolk sac; and the differentiation of the trophoblast into mononucleated cytotrophoblasts and multinucleated syncytiotrophoblasts (figure 3). Although mouse and human embryos develop characteristically different morphologies, Shahbazi and colleagues [23] went on to show that they have their origins in similar cellular events. Just as with mouse ES cells, the Zernicka-Goetz group now demonstrate, via three-dimensional culture of human ES cells or induced pluripotent stem cells, that it is possible to recapitulate the first morphogenetic changes of EPI mediated by cell polarization and cavitation. Thus, the critical remodelling events at this stage of human development are autonomous, highlighting their remarkable and unanticipated self-organizing properties.

**Figure 3.**
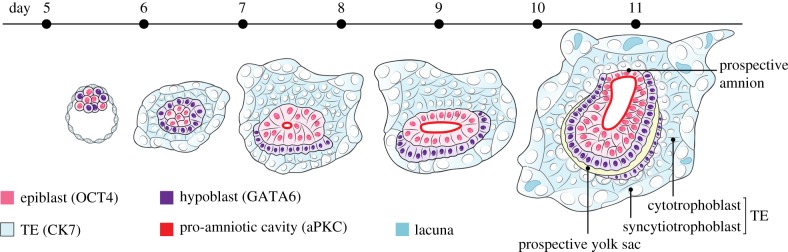
Stages in the development of human embryos cultured *in vitro* to 14 days. Taken from Shahbazi *et al*. [23] and kindly provided by the authors.

Together, these studies signal the potential for developing rational ways of differentiating human pluripotent cells into specific cell types for regenerative medicine. Importantly, they also open a new chapter in embryology. In particular, the work on human embryos establishes a model that will aid our understanding of early pregnancy loss in humans. Almost half of all fertilized eggs die spontaneously before a woman knows she is pregnant and thereafter as many as 20% of pregnancies fail within the first seven weeks before a heartbeat can be detected. These spontaneous deaths, together accounting for 60–70% of all embryos, are largely due to defects in development. It is important to understand how these early stages of development take place in order to be able to predict when developmental defects are likely to arise. However, currently there are ethical limits to what can be done experimentally. In the two studies reported to date [22,23], embryo culture was stopped before 14 days, prior to gastrulation, an internationally agreed limit for the culture of human embryos *in vitro*. There is now a strong argument for extending this limit to be able to study key developmental events not only preceding but also following gastrulation. The public debate on this has already begun but its resolution will take some time. Meanwhile we have only begun to explore the developmental processes leading up to this time and undoubtedly the coming years will lead us to greater understanding of the interactions between the different cell types of the implanting human embryo that shape our identities.
